# Acute Respiratory Infections among Under-five Children Admitted in a Tertiary Hospital of Nepal: A Descriptive Cross-sectional Study

**DOI:** 10.31729/jnma.6889

**Published:** 2022-01-31

**Authors:** Radha Bhurtel, Ram Prasad Pokhrel, Balkrishna Kalakheti

**Affiliations:** 1 Department of Nursing, College of Medical Sciences Teaching Hospital, Bharatpur, Nepal; 2 Department of Pediatrics, College of Medical Sciences Teaching Hospital, Bharatpur, Nepal

**Keywords:** *anemia*, *malnutrition*, *pneumonia*, *respiratory tract infections*

## Abstract

**Introduction:**

Acute respiratory infection is a major cause of mortality and morbidity among under-five children in developing countries. Children under five years of age are most vulnerable to various common but treatable conditions. The objective of this study is to find the prevalence of acute respiratory infections among under-five hospitalized children in a tertiary hospital of central Nepal.

**Methods:**

This was a descriptive cross-sectional study conducted in a tertiary hospital of Nepal from January 2018 to December 2019. Ethical approval was taken from the Institutional review committee (Reference No: 2020-073). Convenience sampling technique was used. Data was entered in the Microsoft excel sheet, then extracted and analyzed in the Statistical package of Social Sciences version 20. Point estimate at 95% Confidence Interval was calculated along with frequency and percentage and presented in tables and figures.

**Results:**

Among 660 children in our study, the prevalence of acute respiratory infection among hospitalized under-five children was 242 (36.67%) (32.99-40.34 at 95% Confidence Interval). Fever and cough were the most common presenting complaints among these children seen in 196 (81%) and 185 (76%) respectively. Stunting and wasting were seen in 37 (15%) and 26 (10.7%) of these children with acute respiratory infection while 6 (2.5%) of them were found overweight.

**Conclusions:**

Acute respiratory infection is the most common reason for hospital admission among children under five years of age and the prevalence was high as compared to the standard study. The chief complaints are fever and cough.

## INTRODUCTION

Nepal's under-five mortality rate decreased by approximately 57%, from 91 deaths per 1000 live births in 2001 to 39 in 2016.^1^ However, under-five mortality in Nepal still remains higher than the Sustainable Development Goal (SDG) target of 20 per 1000 live births.^1^

Children under five years of age are most vulnerable to various common but treatable conditions.^2,3^ Acute respiratory infection (ARI) which includes both upper respiratory tract infections (URTI) and lower respiratory tract infection (LRTI), accounts for up to 50% of hospital visits in children.^4^ URTI such as common cold, pharyngitis, tonsillitis and otitis media also peak in this age. Pneumonia is the leading cause of mortality and morbidity in under-five children globally but its prevalence varies across the globe.^5-7^

Therefore, this study was conducted with the aim to find the prevalence of acute respiratory infections among hospitalized children under five years of age.

## METHODS

A descriptive cross-sectional study was conducted at Department of Pediatrics, College of Medical Sciences Teaching Hospital (COMSTH), Bharatpur, Nepal. Data was collected retrospectively from medical records of all the patients admitted from January 2018 to December 2019 during one-month period, September 2020. The ethical approval was obtained from COMSTH-IRC (Ref. 2020-073). Children between 2 months to 5 years of age were admitted to the pediatric ward through the emergency department, OPD or transferred from PICU were included. Medical records of incomplete information were excluded.

Convenience sampling technique was used. The sample size was estimated using the following formula:

n = Z^2^ × (p × q) / e^2^

  = (1.96)^2^ × (0.5) × (0.5) / (0.04)^2^

  = 600

Where,

n= required sample size
Z= 1.96 at 95% Confidence Interval
p= prevalence of acute respiratory infections among under-five hospitalized children in a tertiary hospital taken as 50% for maximum sample size calculation
e= margin of error, 4%

Adding 10% non-response rate, the total sample size was 660.

Hence, during these two years, 660 children belonging to the under-five age group were included in this study. The patient records of all of these children were used for the study.

A two-part semi-structured questionnaire was used to collect the data which included socio-demographic characteristics (age, gender, height and weight), and questionnaire related to morbidity. Patient record files were reviewed after taking verbal permission from the head of the department of pediatrics and medical record section in-charge.

In this study, acute respiratory infections were defined based on chief complaints, clinical examination and investigations including chest x-ray and included both upper and lower respiratory tract infections. Stunting and wasting were defined by measurement of height for age and weight for height respectively when Z score was less than minus 2 standard deviation (-2SD). These were graded as moderate and severe as per the WHO definition.^8^ Overweight was defined when weight for height Z score was more than 2 standard deviations above median (+2SD).^1^ Anemia in children was defined as hemoglobin value less than 110 mg/dl. Severity of anemia was graded as mild, moderate or severe.^8^

The data entered in the Microsoft Excel sheet. It was then analyzed using Statistical Package for the Social Sciences version 20. The result was obtained in frequency and percentage and presented in the tables and figures.

## RESULTS

During the study period, 660 patients below five years of age were admitted to the pediatric department. Study shows that the prevalence of ARI was 242 (36.67%) (32.99-40.34 at 95% Confidence Interval) among hospital admitted children. Almost two-thirds of these children were admitted from the emergency department and the rest from OPD.

Five most common clinical symptoms at presentation were fever, cough, running nose, difficulty breathing, and noisy breathing respectively (Table 1).

**Table 1:** Main presenting symptoms at admission (multiple responses) (n = 242).

Presenting Complaints	n (%)
Fever	196 (81)
Cough	185 (76)
Running nose	62 (26)
Loose stool	20 (8)
Vomiting	38 (16)
Difficulty breathing	58 (24)
Noisy breathing	32 (13)
Decreased feeding	7 (3)
Fast breathing	6 (2.5)

**Figure 1:**
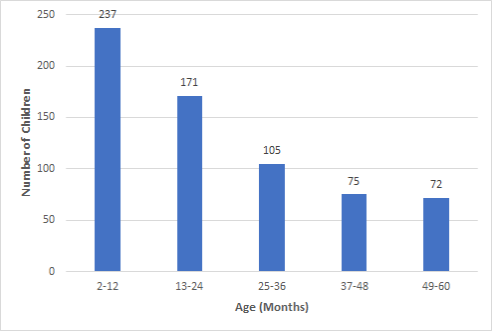
Age distribution of children (n= 660).

Most of the children with ARI were in the first year of life 113 (47%) followed by second 60 (25%) and third year 29 (12%) of life respectively with median age of 15 months (IQR: 30-8=22) (Figure 1).

**Figure 2:**
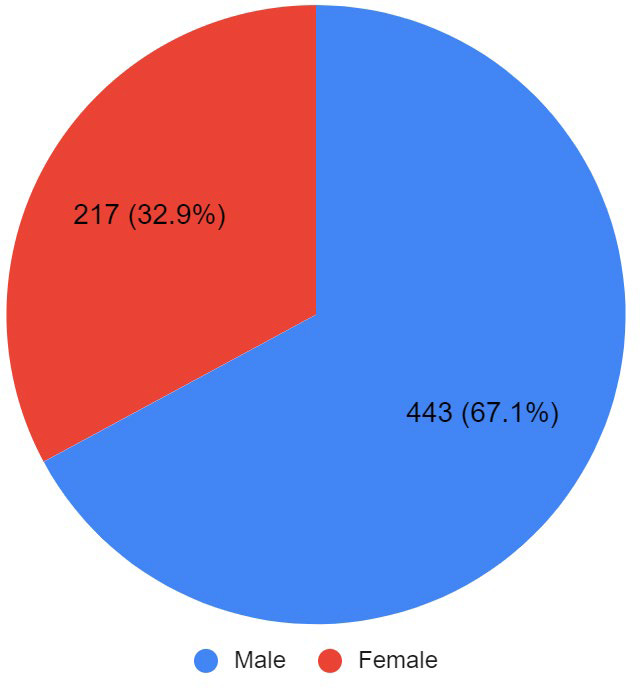
Gender Distribution (n= 660).

Majority of these children were male 172 (71%)(Figure 2). Median duration of hospital stay was 4 days (IQR: 5-2=3) with most of them 201 (83%) staying in hospital for 2-7 days. Twenty six (11%) children were discharged the other day of admission while only 15 (6%) of children stayed for more than 7 days in the hospital.

Stunting and wasting were seen in 37 (15%) and 26 (10.7%) of children while 6 (2.5%) of them were found overweight. Anemia was observed in almost 132 (54.5%) of the children, most of which were mild to moderate in severity (Table 2).

**Table 2:** Nutritional Profile of Under five children admitted in the Hospital with ARI (n = 242).

Nutritional status	n (%)
Stunting (Weight/age)	
Normal	205 (85)
Moderate	37 (15)
**Wasting (Weight/Height)**	
Normal	210 (87)
Moderate PEM	18 (7.4)
Severe PEM	8 (3.3)
Overweight	6 (2.4)
**Anemia (Hb in g/dl)**	
Severe (<7)	5 (2)
Moderate (7-9.9)	69 (28.5)
Mild (10-10.9)	58 (24)
Normal (≥11)	110 (45.5)

The mean hemoglobin was 10.64±1.53 and the range was 15-5.9g/dl.

## DISCUSSION

The present study revealed that the prevalence of acute respiratory infections in hospitalized children was 242 (37%) which makes it the most common reason for hospital admission in children under five year of age. This finding is in agreement with other studies done in community and hospital.^9,10^ This was higher than the prevalence of 2.4% in NDHS 2016.^1^ The same study states that 34% of children with symptoms of ARI sought treatment or advice from pharmacies which points towards the possibility of cases being under reported.

The most common presenting complaints among children with ARI, at the time of hospital admissions were fever and cough similar to study by Koirala R.^11^

In this study, the most common age of hospital admission was infancy. However, the number of children requiring hospital admission decreased as they grew older. Such findings were similar to the study done in Kanti Children Hospital (KCH).^12^

Current study shows that male children were predominantly admitted compared to females. Similar findings were found in the study conducted by Joshi, et al.^12^ and others.^13,14^ This could be due to higher acceptance of parents to admit their sons compared to their daughters^15^ but could also be due to the variable gender composition of the study population.^1^

The study also found that most of the children stayed in hospital for 2-7 days (83%), followed by those who were discharged after 1 day (11%). This was similar to the findings from Lumbini Zonal Hospital and Nepal Medical College where most of the patients were discharged in less than 7 days.^7,16^

This study revealed stunting in 37 (15%) children which is less than the prevalence in NDHS 2016 (35.8%) and prevalence in Bagmati Province (29%).^1,17^ However, wasting 26 (10.7%) appears comparable to the result of NDHS 2016 and also findings by Ujunwa , et al.^1,18^ Prevalence of wasting has fluctuated in Nepal in recent years, from 11% in 2001 to 13% in 2006 to 10% in 2016.^1^ In a community-based study conducted in a village near to the current study place, stunting and wasting among children below five years was 37.3% and 25.7% respectively,^19^ which is higher than the findings in this study. This means that the situation of malnutrition might be worse in the community than seen in this study.

The number of children who were overweight for their age was twice more prevalent 6 (2.5%) than noted in the national survey (1%).^1^ This might be due to the affluent population living in and around Chitwan who form the majority of the study population. On the contrary, a greater number of children visiting clinic in Kathmandu were found overweight compared to the current study.^20^

Anemia has also been stated as one of the risk factor for ARI among under five children^21^ as it potentiates infection by weakening the immune system.^22^ In this study, anemia was found in 132 (54.5%) of children which is similar to findings by Joshi, et al.^23^ However, Sinha, et al. found quite lower prevalence of anemia (28%) among hospitalized children under five years of age in western Nepal.^24^ On contrary to all these, Nepal National Micronutrient Status Survey (NNMSS) 2016 noted anemia only in 19% of children in this age group.^25^ Higher prevalence of anemia in children from cities like Kathmandu,^26^ Bhairawa^24^ and this study from Bharatpur compared to NNMSS 2016 demands further studies.

This was a single center retrospective study. So, a multi-centric prospective study could project the actual prevalence of the condition.

## CONCLUSIONS

ARI is the most common illness among hospitalized children under five years of age. Among them, the most common presenting complaints are fever and cough. Stunting, wasting and anemia are common among these children.
